# Inducible Clindamycin Resistance in *Staphylococcus aureus:* Reason for Treatment Failure

**DOI:** 10.4103/0974-777X.52989

**Published:** 2009

**Authors:** KE Vandana, Jyothsana Singh, M Chiranjay, Indira Bairy

**Affiliations:** *Department of Microbiology, Kasturba Medical College, Manipal, Udupi, Karnataka - 576 104, India*

Sir,

*Staphylococcus aureus* is a versatile human pathogen causing infections ranging from relatively mild skin and soft tissue infection to life threatening sepsis, pneumonia and toxic shock syndrome. It facilitates disease by its propensity to develop resistance to multiple antibiotics that complicates the treatment well exemplified by MRSA, leaving few therapeutic options. This has led to renewed interest in the usage of Macrolide-Lincosamide-StreptograminB antibiotics to treat *S. aureus* infections.[1,2] Macrolide resistance in these organisms is mainly mediated by two mechanisms; MLS type B (MLSb) or efflux mechanism phenotypes. Expression of MLSb phenotype may be either constitutive or inducible in presence of low levels of inducers such as erythromycin. The latter results in increased resistance to MLS class antibiotics such as clindamycin.[3] Clinical failures can occur if resistance mechanisms to these drugs are inadequately tested in the laboratory. Here we present a study to distinguish different resistant phenotypes among staphylococci by D test, a simple and reliable disc induction test.

A total of 112 isolates of *S. aureus* from various clinical specimens were studied for the presence of inducible clindamycin resistance from January through June 2008. Antimicrobial susceptibility was tested for anti-staphylococcal drugs on Mueller Hinton Agar plate by the disc diffusion method. Methicillin resistance was tested using 30μg cefoxitin disc. All isolates were tested for inducible clindamycin resistance (iMLSb phenotype) using D test.[4] Clindamycin (2μg) and erythromycin (15μg) discs were placed 15 mm apart from edge to edge. Plates were read after 18–24 hrs incubation at 37°C for flattening of the clindamycin zone adjacent to erythromycin disc (D shaped zone) suggestive of inducible resistance to clindamycin.

Of the 112 isolates of *S. aureus*, 73 (65%) were methicillin sensitive (MSSA) while 39 (34.5%) were methicillin resistant (MRSA). Among MSSA, 7 (9.5%) strains were found to exhibit inducible clindamycin resistance (iMLSb phenotype), one strain was resistant to both clindamycin and erythromycin (constitutive resistance, cMLSb phenotype), 41 (56.1%) strains were of MS phenotype and 24 (32.8%) were sensitive to both antibiotics. On the other hand, among MRSA strains, 19 (48.7%) belonged to iMLSb phenotype, 12(30.7%) MS phenotype, 2 (0.05%) cMLSb phenotype and 5 (12.8%) susceptible to both antibiotics [Table 1].

**Table 1:** MLSb resistant phenotypes among *S. aureus*

Phenotypes	MSSA, n(%)	MRSA, n(%)	Total, n(%)
ER-R, CL-S, D+ (iMLSb phenotype)	7 (9.5)	19 (48.7)	26 (23.2)
ER-R, CL-R (cMLSb phenotype)	41 (5.61)	12 (30.7)	53 (47.3)
ER-R, CL-S, D− (MS phenotype)	1 (0.01)	2 (0.05)	3 (2.67)
ER-S, CL-S	24 (32.8)	5 (12.8)	29 (25.8)
Total, n(%)	73 (65.5)	39 (34.5)	112

ER: Erythromycin, CL: Clindamycin, R: Resistant, S: Susceptible.

Accurate susceptibility data is the important factor in appropriate therapy decisions. In the light of the restricted antibiotic range available for the treatment of MRSA infections, clindamycin should be considered as part of the treatment regimen in managing serious soft tissue infections. However D test or disc induction method must be implemented in routine clinical laboratories to discriminate between inducible clindamycin resistance and clindamycin susceptibility. Isolates that are erythromycin resistant but clindamycin susceptible should not be reported so unless tested for iMLSb resistance *in vitro*. Thus it is recommended that clindamycin therapy must be avoided for staphylococcal isolates that display iMLSb resistance in spite of low clindamycin MIC. The proportion of staphylococci with *in vitro* inducible clindamycin resistance may vary by hospital, geographic region, bacterial strain and methicillin susceptibility.[5] iMLSb resistance is a significant problem in *S. aureus* isolates, more so in MRSA as found in our study. D test can be used as a simple, auxiliary and reliable method to delineate inducible and constitutive clindamycin resistance in routine clinical laboratories. Misclassification of isolates with iMLSb resistance without D test would lead to treatment failure.
